# P21-PARP-1 Pathway Is Involved in Cigarette Smoke-Induced Lung DNA Damage and Cellular Senescence

**DOI:** 10.1371/journal.pone.0080007

**Published:** 2013-11-11

**Authors:** Hongwei Yao, Isaac K. Sundar, Vera Gorbunova, Irfan Rahman

**Affiliations:** 1 Department of Environmental Medicine, Lung Biology and Disease Program, Rochester, New York, United States of America; 2 Department of Biology, University of Rochester Medical Center, Rochester, New York, United States of America; University of Alabama-Birmingham, United States of America

## Abstract

Persistent DNA damage triggers cellular senescence, which may play an important role in the pathogenesis of cigarette smoke (CS)-induced lung diseases. Both p21^CDKN1A^ (p21) and poly(ADP-ribose) polymerase-1 (PARP-1) are involved in DNA damage and repair. However, the role of p21-PARP-1 axis in regulating CS-induced lung DNA damage and cellular senescence remains unknown. We hypothesized that CS causes DNA damage and cellular senescence through a p21-PARP-1 axis. To test this hypothesis, we determined the levels of γH2AX (a marker for DNA double-strand breaks) as well as non-homologous end joining proteins (Ku70 and Ku80) in lungs of mice exposed to CS. We found that the level of γH2AX was increased, whereas the level of Ku70 was reduced in lungs of CS-exposed mice. Furthermore, p21 deletion reduced the level of γH2AX, but augmented the levels of Ku70, Ku80, and PAR in lungs by CS. Administration of PARP-1 inhibitor 3-aminobenzamide increased CS-induced DNA damage, but lowered the levels of Ku70 and Ku80, in lungs of p21 knockout mice. Moreover, 3-aminobenzamide increased senescence-associated β-galactosidase activity, but decreased the expression of proliferating cell nuclear antigen in mouse lungs in response to CS. Interestingly, 3-aminobenzamide treatment had no effect on neutrophil influx into bronchoalveolar lavage fluid by CS. These results demonstrate that the p21-PARP-1 pathway is involved in CS-induced DNA damage and cellular senescence.

## Introduction

Cigarette smoke (CS) is an important risk factor in chronic inflammatory pulmonary diseases including chronic obstructive pulmonary disease (COPD), due to increased inflammation, oxidative stress, apoptosis/proliferation, and premature senescence/aging [1], [2]. Recently, we have shown that CS exposure causes premature senescence in lung cells, leading to airspace enlargement and lung function decline [3]. Persistent or severe DNA damage is able to trigger cellular senescence [4]–[6]. Indeed, increased DNA damage is observed in lungs of patients with COPD [7]–[12]. However, the molecular mechanism of CS-induced DNA damage and subsequent lung cellular senescence is unknown.

Accumulating evidence has shown that p21^CDKN1A^ (p21), the first identified inhibitor of cyclin/cyclin-dependent kinase complex, participates in the DNA damage response [13]. We, and others, have shown that CS increased the level of p21 protein in lung macrophages and epithelial cells *in vitro* and in mouse lungs *in vivo* [3], [14]–[16]. Poly(ADP-ribose) polymerase 1 (PARP-1), an NAD^+^-dependent ADP-ribosyltransferase 1, is known as a damage sensor, which binds to damaged DNA and thereby promotes the cellular response to DNA single-strand breaks and double-strand breaks (DSB) [17], [18]. For example, PARP-1-mediated PARylation promotes non-homologous end joining (NHEJ) through recruitment or retention of repair factors at DSB sites [19]. Recent studies have shown a physical association between p21 and PARP-1 [20], implicating an involvement of p21-PARP-1 pathway in DNA damage and repair. We, therefore, hypothesized that CS causes DNA damage and premature senescence through p21-PARP-1 signals. To test this hypothesis, we exposed the p21 knockout (p21^-/-^) and C57BL/6J wild-type (WT) mice to CS for 3 days and determined the role of p21 in DNA damage and repair. Furthermore, a selective PARP-1 inhibitor 3-aminobenzamide (3-AB) was administered in p21^-/-^ and WT mice to study the role of p21-PARP-1 in CS-induced DNA damage and cellular senescence.

## Materials and Methods

### Ethics statement

All experimental protocols were performed in accordance with the standards established by the United States Animal Welfare Act, as set forth by the National Institutes of Health guidelines. The research protocol for these studies was approved by the University of Rochester Committee on Animal Research.

### Mice and 3-AB administration

The p21^-/-^ mice was described previously [3], [16], which were obtained from Dr. Michael O'Reilly (University of Rochester) who backcrossed them 10 generations to C57BL/6J after receiving from Dr. Philip Leder at Harvard Medical School (Boston, MA). 3-AB (10 mg/kg, Calbiochem) was administered through intraperitoneal injection at 2 h prior to CS exposure daily for 3 d [21]. Control mice were intraperitoneally injected with saline.

### CS exposure

Eight to ten weeks old mice were used for CS exposure as described previously [16], [22]. The research grade cigarettes (3R4F, University of Kentucky) were used to generate smoke, and mice were exposed to CS according to the Federal Trade Commission protocol (1 puff/min of 2-s duration and 35-ml volume) with a Baumgartner-Jaeger CSM2072i automatic CS generating machine (CH Technologies). The smoke concentration was set at a value of ∼300 mg/m^3^ total particulate matter by adjusting the flow rate of the diluted medical air, and the level of carbon monoxide in the chamber was ∼350 ppm [22]. Mice received two 1 hour exposures (one hour apart) daily for 3 consecutive days, and were sacrificed at 24 h post-last exposure. Control mice were exposed to filtered air in an identical chamber according to the same protocol described for CS exposure.

### Bronchoalveolar lavage (BAL)

The mouse lungs were lavaged three times with 0.6 ml of 0.9% NaCl after intraperitoneal injection of pentobarbiturate (100 mg/kg body weight) [22]. The BAL cell pellet was resuspended in 1 ml of 0.9% NaCl, and the total cell number was determined by counting on a hemocytometer. Differential cell counts (minimum of 500 cells per slide) were performed on cytospin-prepared slides (Thermo Shandon) stained with Diff-Quik (Dade Behring).

### Immunohistochemical staining of proliferating cell nuclear antigen (PCNA)

The PCNA staining was performed using a PCNA kit (Invitrogen, Camarillo, CA). The deparaffinized and rehydrated lung sections were exposed to 3% H_2_O_2_ in methanol for 30 min to quench endogenous peroxidase activity after antigen retrieval using the citrate buffer (0.01 M, pH 6.0). Nonspecific binding of antibodies on the tissue sections were blocked by incubating the sections with 5% normal goat serum in PBS for 30 min. Lung tissue sections were incubated with a primary PCNA antibody at a titer of 1∶100 overnight at 4°C. After being washed, sections were incubated with secondary antibody biotinylated anti-rabbit Ig (DAKO Corp.) for 1 h, and DAB (DAKO) was used as peroxidase substrate. The counterstaining with hematoxylin was performed before examination under a light microscope.

### Preparation of whole cell lysate

The preparation of whole cell lysate from lung tissue was described previously [22]. Briefly, lung tissue (100 mg) was mechanically homogenized with 0.5 ml of radioimmunoprecipitation assay buffer, and the tissue homogenates were kept on ice for 45 min to allow complete cell lysis. Following centrifugation at 13,000 *g* in an eppendorf tube for 5 min, the supernatant was collected as whole cell lysate. Protein level in samples was measured with a BCA kit (Pierce).

### Immunoblot

Protein samples were separated on a 6%–10% sodium dodecyl sulfate- polyacrylamide gel electrophoresis, and separated proteins were electroblotted onto nitrocellulose membranes (Amersham). The membranes were blocked for 1 h at room temperature with 5% BSA, and then probed with a 1∶400–1∶1000 diluted antibodies of anti-p21, anti-poly(ADP-ribose) (pADPr, PAR), anti-PCNA (Santa Cruz), anti-Ku70, anti-Ku80 (Abcam), and anti-γH2AX (Millipore), anti-PARP-1, and anti-GAPDH (Cell Signaling). After three washing steps (10 min each), the levels of protein were detected using secondary antibody (1∶5,000 dilution in 2.5% BSA in PBS containing 0.1% Tween (v/v) 20 for one hour) linked to horseradish peroxidase (Dako), and bound complexes were detected using ECL method (Perkin Elmer). Equal loading of the samples was determined by quantitation of proteins and by reprobing membranes for GAPDH.

### Senescence-associated β-galactosidase (SA-β-gal) activity assay

The SA-β-gal staining was performed in OCT-embedded frozen lung tissues using a commercial kit (Cell Signaling) [3]. Briefly, the lungs were fixed in 2% formaldehyde containing 0.2% glutaraldehyde for 15 min. After washed with PBS, the samples were incubated at 37°C for 24 h in the staining solution (pH 6.0) containing 1 mg/ml of X-gal. The cells with blue color are SA-β-gal positive. The SA-β-gal activity in lung homogenate was also measured by the rate of conversion of 4-methylumbelliferyl-β-d-galactopyranoside to the fluorescent hydrolysis product 4-methylumbelliferone at pH 6.0 [3]. Briefly, the lung tissues were homogenized in the lysis buffer (pH 6.0), and kept on ice for one hr. The lysates were centrifuged for 5 min at 12,000 *g*, and the supernatant was mixed with 2× reaction buffer containing 1.7 mM of 4-methylumbelliferyl-β-d-galactopyranoside, which was placed into a 37°C water bath for 3 h. Finally, 50 µl of the reaction mix was added to 500 µl of 400 mM sodium carbonate stop solution (pH 11.0), which was read at 150 µl per well in a 96-well plate using a SpectrumMax M5 plate reader (Molecular Devices) with excitation at 360 nm, emission at 465 nm, 40 µs integration, and gain held constant at 46. Normalized SA-β-gal activity is expressed as observed fluorescence divided by micrograms of total protein.

### Statistical analysis

Statistical analysis of significance was calculated using one-way Analysis of Variance (ANOVA) for multigroup comparisons using STATVIEW. The results are shown as the mean ± SEM. The *P*<0.05 is considered as statistically significant.

## Results

### CS exposure causes DNA damage and cellular senescence along with increased p21 level

**Figure 1:**
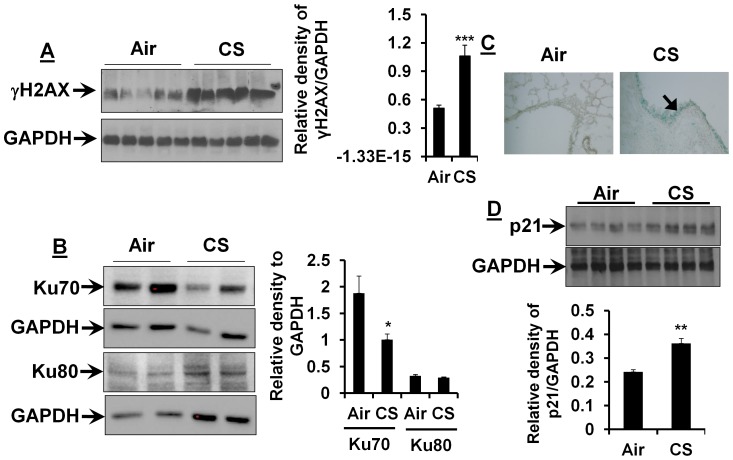
CS exposure induces DNA damage and cellular senescence associated with increased p21 level in mouse lungs. CS increased γH2AX level (**A**), but reduced the levels of Ku70 in mouse lungs (**B**). Both SA-β-gal activity (**C**) and p21 level (**D**) were increased in lungs of CS-exposed mice. Gel pictures shown are representative of at least 3 separate mice. Relative density ratio is indicative of results after normalizing to corresponding GAPDH. Data are shown as mean ± SEM (n = 3 to 6 per group). Original magnification, ×200. ^*^ *P*<0.05, ^**^ *P*<0.01, ^***^ *P*<0.001 *vs* air-exposed mice.

An early cellular response to DSB is the rapid phosphorylation of H2AX at Ser139 (γH2AX), which is a sensitive molecular marker for DNA damage [23], [24]. To determine whether CS causes DNA damage, we performed the immunoblotting for γH2AX in mouse lungs. As shown in **Figure 1A**, the level of γH2AX was significantly increased in mouse lungs exposed to CS for 3 days. We also observed that the levels of NHEJ proteins Ku70, but not Ku80, were reduced in CS-exposed mouse lungs (**Figure 1B**). Persistent DNA damage has been shown to cause cellular senescence [4], [5], [25]. Therefore, we determined cellular senescence via measuring SA-β-gal activity and p21 levels; and found that CS caused an increase in SA-β-gal activity and p21 level in mouse lungs (**Figure 1C and 1D**). These findings suggest that CS-induced DNA damage and cellular senescence are associated with increased p21 level.

### Deletion of p21 attenuates CS-induced DNA damage and NHEJ repair impairment

**Figure 2:**
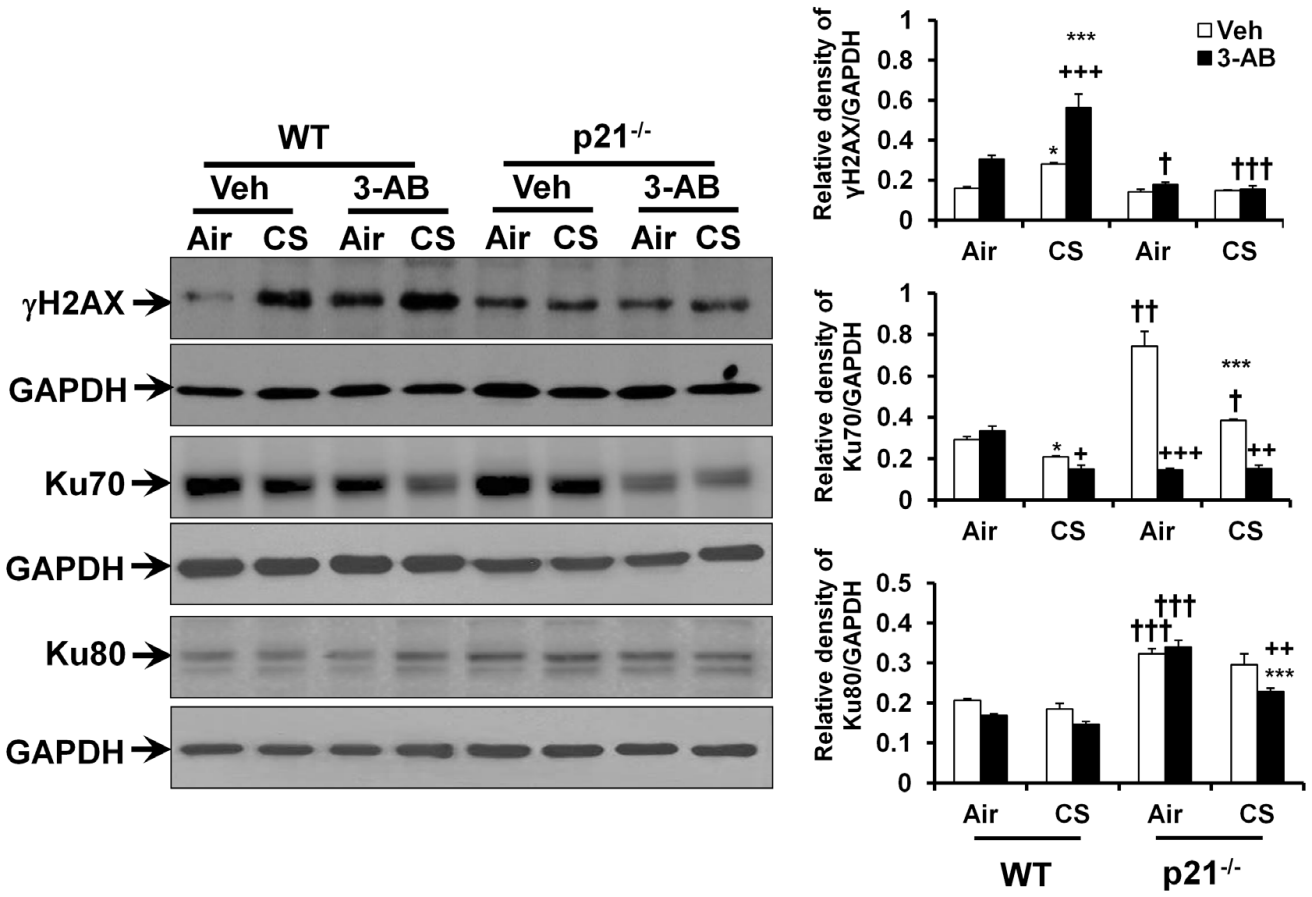
p21 deletion decreases the level of γH2AX but augments the levels of Ku70 and Ku80 in mouse lung, which was affected by PARP-1 inhibitor. γH2AX level was decreased, whereas the levels of Ku70 and Ku80 were augmented in lungs of p21^-/-^ mice compared to WT mice exposed to CS. 3-AB treatment further increased γH2AX level, but reduced the levels of Ku70 in WT mice. Gel pictures shown are representative of at least 3 separate mice. Relative density ratio is indicative of results after normalizing to corresponding GAPDH. Data are shown as mean ± SEM (n = 3per group). ^*^ *P*<0.05, ^***^ *P*<0.001 *vs* air group; ^++^ *P*<0.01, ^+++^ *P*<0.001, *vs* Veh group; ^†^ *P*<0.05, ^††^ *P*<0.01, ^†††^ *P*<0.001 *vs* WT mice.

**Figure 3:**
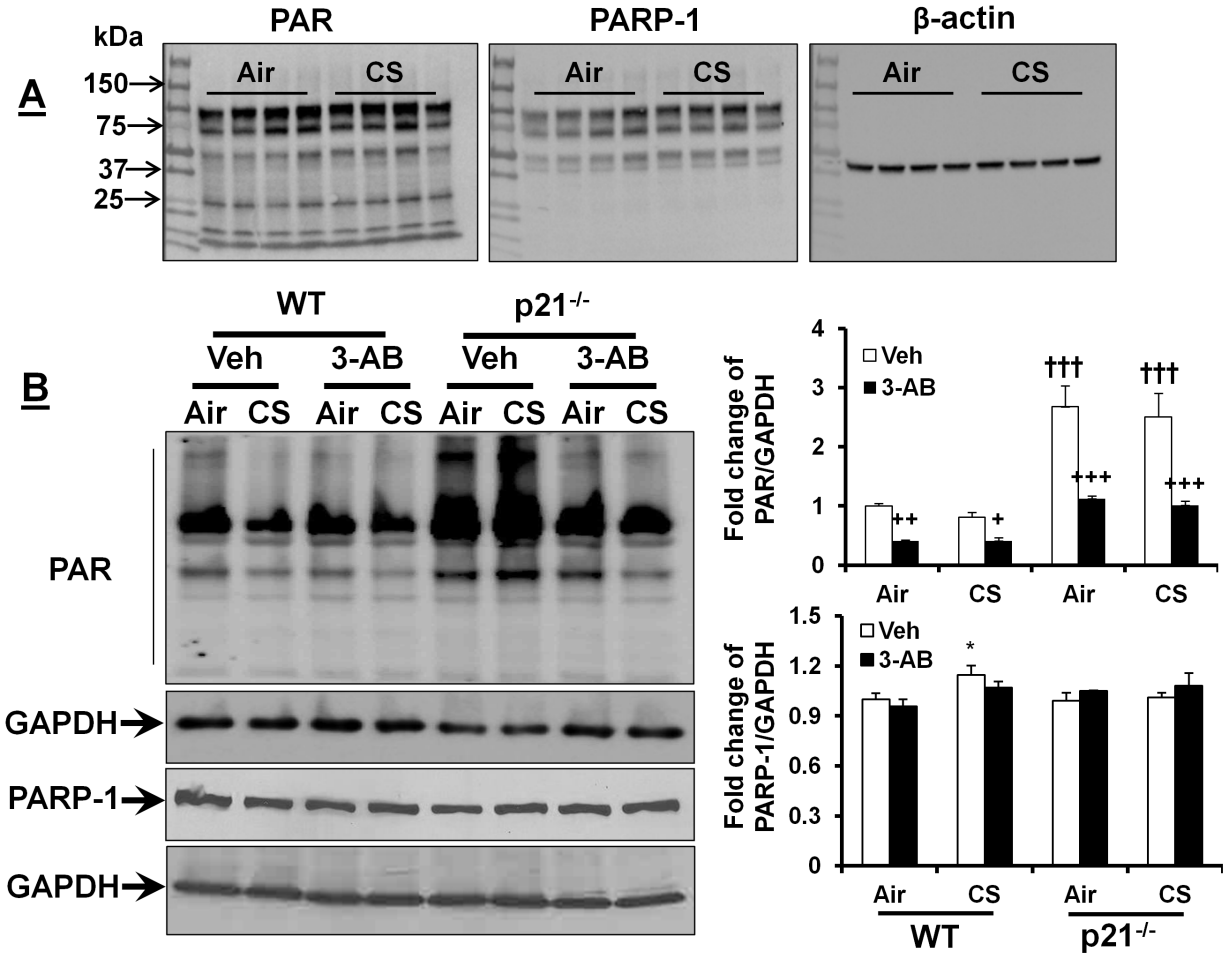
p21 deletion increased the levels of PAR, which was reduced by 3-AB treatment. The levels of PAR and PARP-1 were determined by Western blot in lungs of C57BL/6J (**A**), and p21^-/-^ mice as well as WT littermates (**B**) in response to CS. PAR levels were increased in lungs of p21^-/-^ mice as compared to WT mice. 3-AB treatment reduced PAR level in lungs of both p21^-/-^ and WT mice. Intact PARP-1 level was not altered by either p21 deficiency or 3-AB treatment. Gel pictures shown are representative of at least 3 separate mice. Fold change is indicative of the alteration of PAR and PARP-1 compared with air-exposed and vehicle (Veh)-treated WT mice after normalizing to corresponding GAPDH or β-actin. Data are shown as mean ± SEM (n = 3-13 per group).^ *^ *P*<0.05 *vs* air group; ^+^ *P*<0.05, ^++^ *P*<0.01, ^+++^ *P*<0.001 *vs* Veh group; ^†††^ *P*<0.001 *vs* WT mice.

To further determine the causal role of p21 in CS-mediated DNA damage, the p21^-/-^ and WT mice were exposed to CS for 3 days in order to compare the lung DNA damage response. We found that p21 deletion decreased the level of γH2AX in CS, but not in air-exposed mouse lungs (**Figure 2**). Further, the levels of Ku70 and Ku80 were increased in lungs of p21^-/-^ mice as compared to WT mice regardless of air or CS exposure (**Figure 2**). We then focused on whether p21-mediated DNA damage is associated with the regulation of PARP-1 since a physical interaction between p21 and PARP-1 is reported recently [20]. As expected, p21 deletion increased the level of PAR (reflecting PARP-1 activity) in both air- and CS-exposed mouse lungs, although CS exposure did not have any effect on the level of PAR in WT or p21^-/-^ mice (**Figure 3A and 3B**). The level of full length PARP-1 in lungs was increased by CS exposure in WT but not in p21^-/-^ mice (**Figure 3B**). These results suggest that p21 deletion attenuates CS-induced DNA damage, which is associated with PARP-1 activation.

### PARP-1 inhibitor increases DNA damage and cellular senescence, but has no effect on lung inflammatory response to CS

As shown in **Figures 3**, PARP-1 activation upon p21 deletion was associated with protection against CS-induced DNA damage in mouse lungs. Therefore, we determined the role of PARP-1 in p21-mediated DNA damage by administering a selective PARP-1 inhibitor 3-AB to p21^-/-^ and WT mice prior to CS exposure. As expected, lung PARP-1 activity, which is reflected by PAR level, was inhibited after 3-AB treatment (**Figure 3**). Administration of 3-AB increased the levels of γH2AX in lungs of WT, but did not increase in p21^-/-^ mice exposed to CS (**Figure 2**). 3-AB treatment significantly reduced the levels of Ku70 in lungs of both WT and p21^-/-^ mice exposed to CS (**Figure 2**). Furthermore, the level of Ku80 was decreased by 3-AB treatment in p21^-/-^, but not in WT mice by CS exposure (**Figure 2**).

**Figure 4:**
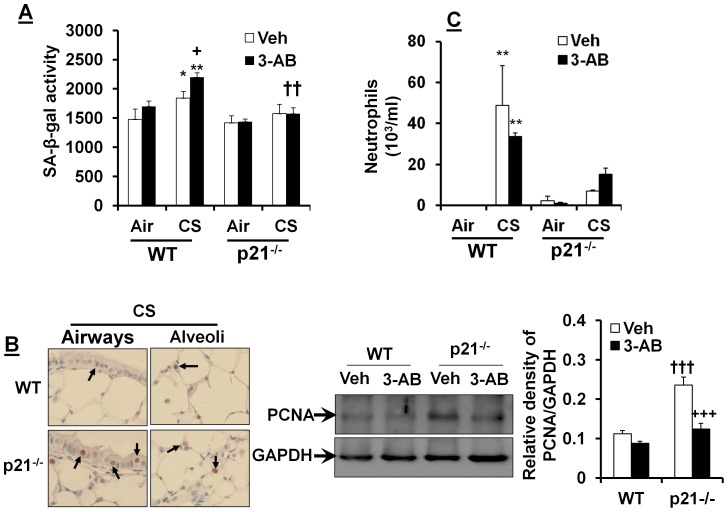
3-AB increases CS-induced cellular senescence, but does not affect neutrophil influx in mouse lungs. 3-AB augmented CS-induced increase in SA-β-gal activity in WT, but not p21^-/-^ mice (**A**). p21 deletion increased the expression of PCNA in lungs as compared to WT mice exposed to CS, which was reduced by 3-AB treatment (**B**). p21 deletion attenuated CS-induced neutrophil influx in BAL fluid, which was not affected by 3-AB (**C**). Original magnification, ×200. Gel pictures shown are representative of at least 3 separate mice. Relative density ratio is indicative of results after normalizing to corresponding GAPDH. Data are shown as mean ± SEM (n = 3-4 per group). ^*^ *P*<0.05, ^**^ *P*<0.01 *vs* air group; ^+^ *P*<0.05, ^+++^ *P*<0.001 *vs* Veh group; ^††^ *P*<0.01, ^†††^ *P*<0.001 *vs* WT mice.

It is known that persistent DNA damage is able to induce cellular senescence [4]–[6], [26]. Therefore, we determined the cellular senescence in response to PARP-1 inhibition by 3-AB; and found that 3-AB treatment increased SA-β-gal activity in lungs of WT, but not in p21^-/-^ mice exposed to CS (**Figure 4A**). Further, p21 deletion increased the expression of PCNA (a proliferation biomarker) in mouse lungs exposed to CS, which was reduced by 3-AB treatment (**Figure 4B**). However, the number of neutrophils in BAL fluid was not altered by 3-AB administration either in p21^-/-^ or WT mice in response to CS exposure, although p21 deletion reduced CS-induced neutrophil influx in BAL fluid (**Figure 4C**). Altogether, our data show that p21 deletion protects against CS-induced DNA damage and cellular senescence via PARP-1 activation.

## Discussion

We have shown that cellular senescence and premature aging play important roles in the development of COPD/emphysema [2], [3]. Persistent DNA damage has been shown to trigger cellular senescence [4], [5]. However, the mechanisms of CS-induced DNA damage and cellular senescence remain unknown. In this study, we found that DNA damage and NHEJ impairment occurred in lungs of CS-exposed mice. Furthermore, p21 deletion reduced DNA damage, but increased NHEJ repair proteins in mouse lungs in response to acute CS exposure. These findings suggest that p21 is an important mediator in CS-induced DNA damage and DNA repair impairment. However, the link between p21 and DNA damage/repair as well cellular senescence is not known.

It has been shown that p21 accumulates rapidly at DNA damage sites [27]–[29], which may be a marker for DNA damage. We found that the levels of both γH2AX (recognition of DNA damage) and p21 were increased in lungs of CS-exposed mice. This is in agreement with the findings that elevation of p21 level in senescent cells correlates with the accumulation of γH2AX [30]. The DSB is the most dramatic form of DNA damage, which is repaired predominantly by NHEJ [31]–[33]. Except for NHEJ pathway of DNA repair, Ku proteins (Ku70 and Ku80) also function as a molecular scaffold, to which other proteins involved in NHEJ can bind [34]. The levels of NHEJ proteins Ku70 were decreased by CS exposure. Hence, CS exposure not only causes DNA damage but also impairs NHEJ repair. It is possible that the regulation of other NHEJ factors (e.g., DNA ligase IV and DNA-PKcs) are also affected in response to CS exposure, which requires further studies. Interestingly, p21 deletion reduced the level of γH2AX, but increased the levels of Ku70 and Ku80 in mouse lungs, suggesting an important role of p21 in CS-induced DNA damage and repair impairment. This is consistent with the findings that p21 causes DNA damage, and inhibits nucleotide excision repair as well as NHEJ via a redox dependent mechanism [35]–[42]. However, recent studies have also demonstrated that p21 protein helps DNA repair and homologous recombination (HR) [41], [43], [44]. The discrepancy among these studies may be due to the different experimental conditions (e.g., low and high levels of DNA damage imposed by oxidative stress) and DNA repair pathways (HR *vs* NHEJ) as well as the use of different cell (*in vitro*) and rodent (*in vivo*) model systems [13], [45].

PARP-1, the most abundant nuclear enzyme of PARP family, catalyzes the formation of the polymer pADPr (PAR) using NAD^+^ as substrate. The level of PAR represents the relative activity of PARP-1. PARP-1 is activated in response to DNA damage, which participates in DNA repair and genomic integrity [17], [18], [46]–[49]. This is in agreement with ours and others' findings that PARP-1 level is increased during CS-induced DNA damage [47]–[51], whereas conflicting results were obtained in other studies [52], [53]. Nevertheless, the role of PARP-1 in DNA damage and repair during the pathogenesis of COPD is implicated [54]. A recent study has shown a physical association between p21 and PARP-1 [20], implicating the regulation of p21-PARP-1 axis in CS-mediated DNA damage. We found that p21 knockout mice showed augmented PARP-1 activity in lungs when compared to WT mice regardless of air or CS exposure. This is corroborated by the findings that p21 negatively regulates PARP-1 activity [20]. Administration of PARP-1 inhibitor 3-AB increased the level of γH2AX, but reduced Ku70 level in mouse lungs exposed to CS. This suggests a positive regulation of PARP-1 in NHEJ DNA repair [19], [55]. We and others have shown that PARP-1 also regulates HR, which is used by cells to accurately repair breaks on both strands of DNA [46], [56], [57]. Hence, PARP-1 may control HR (e.g., RAD51) in response to CS exposure, which needs to be investigated. Overall, PARP-1 activation upon p21 deletion may mediate the protection against CS-induced DNA damage via NHEJ repair. PARP-1 can localize on the promoters, and impact chromatin integrity via PARylation [19], [58], which may regulate the expression of genes and their encoding proteins such as Ku70. This may explain the alteration of Ku70 after PARP-1 inhibitor treatment in mouse lungs.

**Figure 5:**
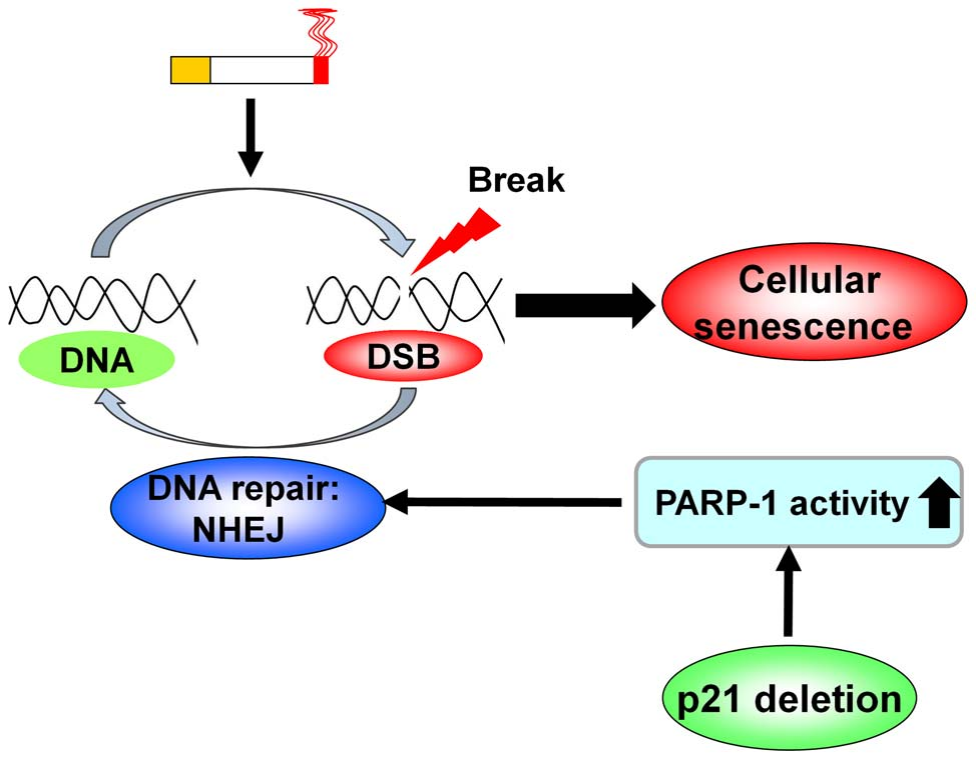
A schematic model showing the role of p21-PARP-1 in acute cigarette smoke (CS)-induced DNA damage and cellular senescence. CS exposure causes DNA damage including double-strand break (DSB). CS exposure also increases the level of p21, and p21 gene deletion augments PARP-1 activity and the levels of non-homologous end joining (NHEJ) proteins to repair damaged DNA. Consequently, CS-induced cellular senescence is attenuated by p21 deletion.

Persistent or severe DNA damage has been shown to trigger cellular senescence [4], [5]. We found increased activity of SA-β-gal and p21 level along with augmented DNA damage in mouse lungs exposed to CS. Deletion of p21 reduced CS-induced DNA damage and cellular senescence [3]. Interestingly, PARP-1 inhibitor further increased CS-induced cellular senescence (i.e., increased SA-β-gal activity) in WT mouse lungs. This is in line with a study showing induction of cellular senescence via inhibition of PARP by veliparib [59]. We noticed that treatment with a PARP-1 inhibitor (3-AB) did not increase lung γH2AX level or SA-β-gal activity as compared to vehicle control in p21^-/-^ mice in response to CS exposure. This may be due to the sufficient endogenous NHEJ or compensatory PARP-1 activity in these mice for repairing damaged DNA. Altogether, p21 deletion augments PARP-1 activity and NHEJ repair, thereby reduces CS-mediated DNA damage and subsequent cellular senescence (**Figure 5**). However, further studies are required to investigate the role of p21-PARP-1 in regulating lung DNA damage and repair as well as cellular senescence during the development of COPD/emphysema.

Senescent cells develop a PARP-1 and NF-κB-associated secretome, and these cells are prone to secrete inflammatory mediators, such as IL-6 and IL-8 [60]. Interestingly, inhibition of PARP-1 by 3-AB did not exhibit any effect on CS-induced neutrophil influx. In fact, the effect of PARP-1 on inflammatory response is controversial. Most of the studies showed the pro-inflammatory effect of PARP-1 in lung inflammation [61]–[63], whereas increased lung inflammation occurred in PARP-1 knockout mice in response to hyperoxia [64]. The discrepancies among these studies are unresolved, which need further investigation.

In conclusion, our findings reveal the important role of p21-PARP-1 axis in regulating CS-induced lung DNA damage and NHEJ impairment as well as cellular senescence. Therefore, both PARP-1 and p21 would be potential targets in intervening CS-induced DNA damage and premature aging as well as subsequent chronic pulmonary diseases, such as COPD.
